# P-1993. Nirmatrelvir/Ritonavir in Immunocompromised Hosts: Use and Outcomes from a Cancer Center

**DOI:** 10.1093/ofid/ofae631.2151

**Published:** 2025-01-29

**Authors:** Darra Drucker, Zahra Kassamali-Escobar, Frank Tverdek, Catherine Liu, Emily A Rosen

**Affiliations:** University of Washington Medicine, Irvine, California; University of Washington Center for Stewardship in Medicine / Fred Hutchinson Cancer Center, Seattle, Washington; Fred Hutchinson Cancer Center, Seattle, Washington; Fred Hutchinson Cancer Center, Seattle, Washington; Fred Hutchinson Cancer Center / University of Washington, Seattle, Washington

## Abstract

**Background:**

Prolonged viral replication of severe acute respiratory syndrome coronavirus 2 (SARS-CoV-2) in immunocompromised (IC) hosts has raised questions about whether treatment duration with nirmatrelvir/ritonavir (nirmatrelvir/r) should be longer than the authorized 5-day course. Data describing nirmatrelvir/r treatment outcomes are limited in the IC population. The primary objective of this study was to assess the efficacy of a nirmatrelvir/r 5-day course as treatment for coronavirus disease 2019 (COVID-19) in IC patients.

**Figure:**
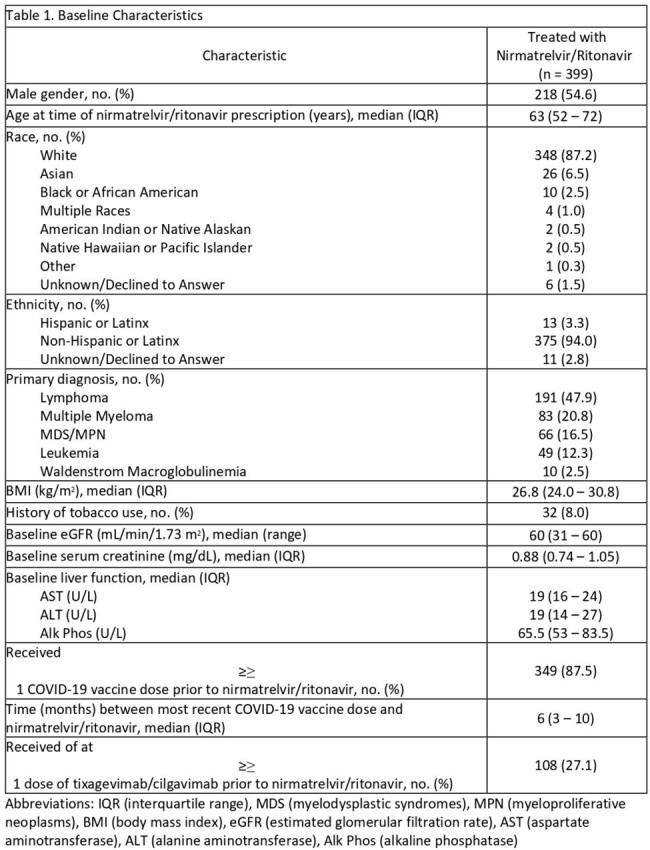

**Methods:**

A retrospective chart review was conducted at Fred Hutchinson Cancer Center (FHCC) of patients ≥ 18 years old with an active hematologic malignancy who received nirmatrelvir/r under Emergency Use Authorization between 12/22/21 - 5/25/23. Patients with an active oncology treatment plan within 3 months (before or after) their nirmatrelvir/r prescription date were included. Patients who received nirmatrelvir/r under Investigational New Drug authorization were excluded. The primary outcome was a composite of all-cause mortality or COVID-19 related hospitalization within 30 days of nirmatrelvir/r. A secondary outcome, persistent COVID-19 infection, was defined as a positive SARS-CoV-2 polymerase chain reaction test 30-90 days after initial positive test.

**Figure:**
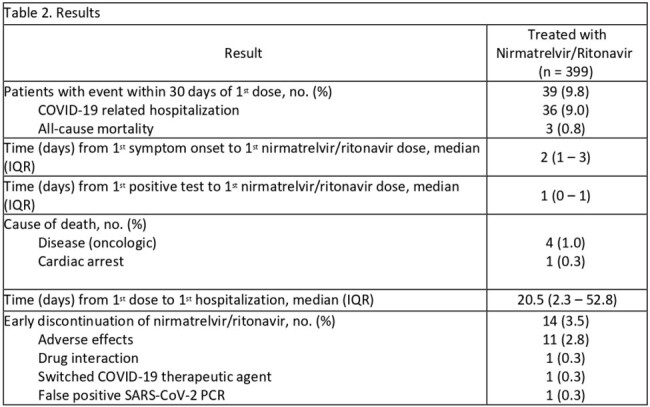

**Results:**

Among 928 patients who received nirmatrelvir/r at FHCC, 399 patients were included. Median age was 63 years, 55% of patients were male, and lymphoma was the most common malignancy, 48% (Table 1). Thirty-day all-cause mortality or COVID-19 related hospitalization occurred in 39/399 (9.8%) of patients. Thirteen patients (3.3%) had persistent COVID-19 infection; 8 had persistent symptoms and 5 required additional treatment. Early discontinuation of nirmatrelvir/r due to adverse effects, most commonly gastrointestinal, occurred in 11 (2.8%) patients (Table 2).

**Conclusion:**

Incidence of the primary outcome was higher in our population compared to rates in nirmatrelvir/r phase 3 trial data and driven by hospitalization rather than all-cause mortality. Persistent infection requiring further treatment occurred in only 1.3% of patients. Additional studies are needed to define optimal treatment duration, including potential indications for use > 5 days in IC hosts.

**Disclosures:**

Frank Tverdek, PharmD, Merck: Advisor/Consultant Catherine Liu, MD, Pfizer: Grant/Research Support

